# Supplementary material for: Complex‐centric proteome profiling by SEC‐SWATH‐MS
Source: Mol Syst Biol. 2019 Jan 14;15(1):e8438. doi: 10.15252/msb.20188438 (PMC6346213; doi:10.15252/msb.20188438)
Supplement: Supplementary file 7 — Dataset EV6 [file MSB-15-e8438-s007.zip › feature_plots_bioplex/P28070.pdf]

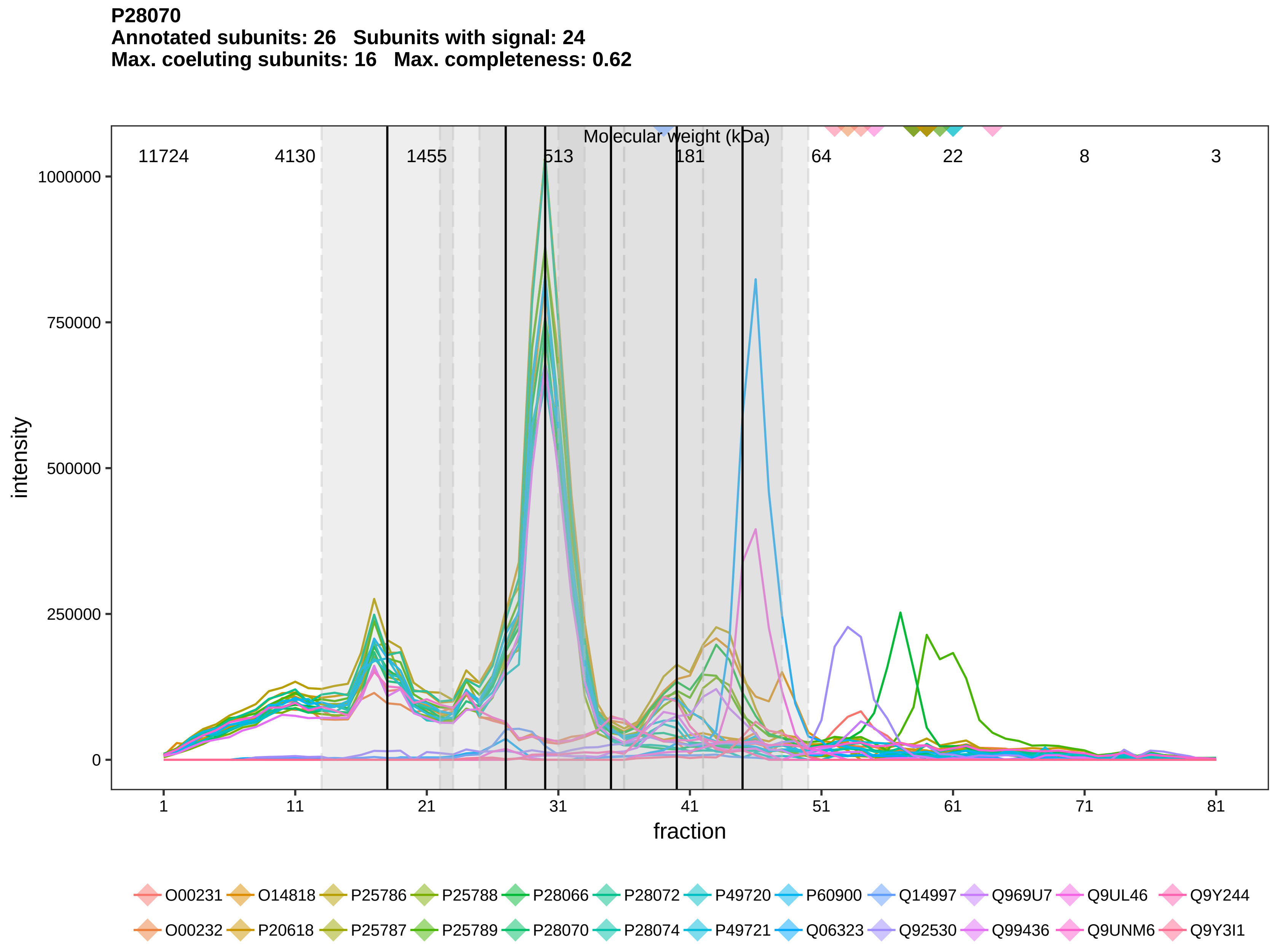

P28070
Annotated subunits: 26 Subunits with signal: 24
Max. coeluting subunits: 16 Max. completeness: 0.62
Molecular weight (kDa)
11724
4130
1455
513
181
64
22
8
3
intensity
1000000
750000
500000
250000
0
1
11
21
31
41
51
61
71
81
fraction
O00231
O14818
P25786
P25788
P28066
P28072
P49720
P60900
Q14997
Q969U7
Q9UL46
Q9Y244
O00232
P20618
P25787
P25789
P28070
P28074
P49721
Q06323
Q92530
Q99436
Q9UNM6
Q9Y3I1
